# Study on Anti-Inflammatory Effects of and Muscle Recovery Associated with Transdermal Delivery of *Chaenomeles speciosa* Extracts Using Supersonic Atomizer on Rat Model

**DOI:** 10.3390/antiox13060702

**Published:** 2024-06-07

**Authors:** Tai-Jung Hsieh, Pin-Yu Chen, Hung-Yi Wang, Chun-Shien Wu, Li-Feng Liu, Kun-Lieh Wu, Shyh-Ming Kuo

**Affiliations:** 1 Department of Electrical Engineering, I-Shou University, Kaohsiung 84001, Taiwan; daito68@gmail.com; 2 Department of Biomedical Engineering, I-Shou University, Kaohsiung 84001, Taiwan; christina64270518@gmail.com; 3 Department of Sports Technology and Leisure Management, I-Shou University, Kaohsiung 84001, Taiwan; wanghy@isu.edu.tw; 4 Center of General Education, I-Shou University, Kaohsiung 84001, Taiwan; wucs@isu.edu.tw; 5 School of Medicine, I-Shou University, Kaohsiung 84001, Taiwan; liulf@isu.edu.tw; 6 YJ Biotechnology Co., Ltd., New Taipei City 105037, Taiwan; bioptik.carlos@gmail.com

**Keywords:** *Chaenomeles speciosa*, oleanolic acid, ursolic acid, supersonic atomizer, anti-inflammation, rat model

## Abstract

Repetitive motion or exercise is associated with oxidative stress and muscle inflammation, which can lead to declining grip strength and muscle damage. Oleanolic acid and ursolic acid have anti-inflammatory and antioxidant properties and can be extracted from *Chaenomeles speciosa* through ultrasonic sonication. We investigated the association between grip strength declines and muscle damage induced by lambda carrageenan (LC) injection and exercise exposure in rats. We also assessed the reparative effects of transdermal pretreatment and post-treatment with *C. speciosa* extracts (CSEs) by using a supersonic atomizer. The half-maximal inhibitory concentration (IC_50_) of CSEs for cells was 10.5 mg/mL. CSEs significantly reduced the generation of reactive oxygen species and inflammatory factors (interleukin [IL]-6 and IL-1β) in in vitro cell tests. Rats subjected to LC injection and 6 weeks of exercise exhibited significantly increased inflammatory cytokine levels (IL-1β, TNF-α, and IL-6). Hematoxylin and eosin staining revealed inflammatory cell infiltration and evident muscle damage in the gastrocnemius muscle, which exhibited splitting and the appearance of the endomysium and perimysium. The treated rats’ grip strength significantly declined. Following treatment with CSEs, the damaged muscles exhibited decreased IL-1β, TNF-α, and IL-6 levels and normal morphologies. Moreover, grip strength significantly recovered. Pretreatment with CSEs yielded an immediate and significant increase in grip strength, with an increase of 180% and 165% occurring in the rats exposed to LC injection and exercise within the initial 12 h period, respectively, compared with the control group. Pretreatment with CSEs delivered transdermally using a supersonic atomizer may have applications in sports medicine and training or competitions.

## 1. Introduction

In sports science, rigorous training and competition can cause fatigue in the musculoskeletal system, resulting in pain, soreness, inflammation, and reduced muscle function. Muscle soreness can arise because of exercise-induced damage to a muscle and its surrounding connective tissue, which can ultimately impair muscle performance [1]. Intensive and prolonged exercise, along with sustained muscle contractions, increase the production of reactive oxygen species (ROS) and induce oxidative stress in skeletal muscles [2]. Elevated levels of ROS can be harmful to cells, contributing to premature muscle fatigue and resulting in muscle damage characterized by structural alterations in muscle tissue, reduced muscle strength, and increased soreness. The recovery process following muscle injury involves a series of critical steps, including degeneration, inflammation, muscle regeneration, and fibrosis [3]. The inflammatory response of musculoskeletal tissue in this process is a key contributor to the pathogenesis of soft tissue disorders [4]. In vitro studies have demonstrated that pro-inflammatory cytokine tumor necrosis factor-α (TNF-α) is a crucial endocrine stimulus contributing to contractile dysfunction in chronic inflammation. Muscle-derived ROS and nitric oxide actively participate in depressing muscle fiber force, potentially leading to muscle atrophy [5]. Elevated systemic levels of TNF-α are associated with reduced muscle strength and mass [6]. Interleukin (IL)-6, a key cytokine involved in low-grade chronic inflammation, is secreted by immune cells in response to tissue infection or damage, and has been implicated in facilitating muscle atrophy [7]. TNF-α and IL-6 are commonly used as markers for assessing the association between inflammation and muscle strength [8]. In addition, transforming growth factor beta (TGF-β), a multifunctional cytokine, acts on skeletal muscles by inhibiting myogenic responses, regulating extracellular matrix remodeling, and stimulating fibrosis [9]. Gumucio et al. reported that inhibiting TGF-β action can result in the rapid recovery of muscle strength in the short term, but may lead to incomplete structural regeneration, ultimately reducing muscle strength over the long term [10]. These findings highlight the critical roles of TGF-β in the regeneration of damaged muscle. In the current study, we considered inflammation to be a natural response to potentially harmful stimuli. Although regular exercise or physical training can serve as a long-lasting, anti-inflammatory therapy that can mitigate inflammatory responses in the muscles, drugs or treatments capable of reducing or eliminating inflammatory factors to facilitate the quick recovery of muscle function and improve exercise performance must be identified to enable further participation in training or competitions.

Oxidative stress is a condition characterized by an imbalance between the cellular production of pro-oxidant molecules and the ability of the antioxidant system to reduce ROS. Studies have indicated that oxidative stress becomes apparent after exercise-induced muscle damage [11]. In such cases, antioxidants play a crucial role in regulating ROS levels through their direct free radical scavenging mechanisms. Because of this, antioxidant supplements are commonly consumed to minimize exercise-induced oxidative stress and to potentially enhance muscle recovery and improve exercise performance [12]. In addition, cold-water immersion is frequently used after strenuous exercise to aid in recovery, reduce muscle soreness, and expedite a return to optimal performance levels [13]. However, its effect on the delayed onset of muscle soreness or performance remains limited and inconclusive. Another option, noninvasive, low-energy laser therapy, is effective at reducing soreness but is expensive, requires off-site application, involves a prolonged course of therapy, and does not provide a long-lasting effect. Additionally, massage therapy offers short-term soreness-reducing benefits; however, it does not lead to a significant recovery of muscle function. In terms of drug treatments, nonsteroidal anti-inflammatory drugs, oral pain relievers, or muscle relaxants may be used as first-line adjuvant medications to alleviate symptoms [14]. Although various treatment methods and strategies have been applied and have produced some positive effects, they have some inherent disadvantages, such as having high costs, offering only short-term relief, and not providing substantial recovery of muscle function.

In addition to exercise-induced muscle damage, the present study considered chemical insults to muscle tissue as a contributor to muscle-related damage, including inflammation. This study used the intramuscular injection of lambda carrageenan (LC) as a model for inflammatory muscle pain or damage resembling myositis in animals [15]. LC induces muscle inflammation leading to primary hyperalgesia, as evidenced by a reduction in grip strength [15]. The current study’s LC model represents acute inflammatory hyperalgesia, with the peak reduction in grip strength occurring at 12–24 h after LC injection. Grip strength typically returns to baseline levels after 48 h. Our use of this animal model enabled us to evaluate new compounds or therapeutic techniques for alleviating muscular inflammation or pain by measuring grip strength.

New treatment methods and strategies involving both chemicals and devices are likely to be developed soon. *Chaenomeles speciosa*, a medicinal plant, contains various chemical components, including oleanolic acid (OA) and ursolic acid (UA). *C. speciosa* further contains flavonoids, anthocyanins, ellagic acid, and dietary fiber. Consequently, the plant exhibits diverse pharmacological effects; it has antioxidant, anti-inflammatory, and antibacterial properties, as well as lipid-lowering and liver-protective capabilities [16]. OA, a natural pentacyclic triterpenoid, possesses antioxidant, anti-inflammatory, and antidiabetic effects [17]. Studies have reported that OA can reduce serum levels and the gene expression of pro-inflammatory cytokines in mice with related insulin metabolic diseases [18]. UA, another natural triterpenoid compound, possesses a range of pharmacological properties, including antioxidant, anti-inflammatory, antibacterial, and antiapoptotic effects [19]. UA has demonstrated promise as an alternative medicine that can be used to treat and prevent conditions such as cancer, obesity and diabetes, liver disease, and muscle sarcopenia [20]. In the current study, we extracted *C. speciosa* through ultrasonic oscillation and isolated OA and UA as targeted chemicals to evaluate their anti-inflammatory effects on lipopolysaccharide (LPS)-induced inflamed C2C12 cells and explore the potential of *C. speciosa* extracts for improving muscle grip strength.

In the context of sports, various strategies for optimizing performance in training and competitions have been explored. Although the oral administration of antioxidant or nutritional supplements is commonly favored in daily training routines, the location, timing, and release profile of these supplements must be controlled to minimize toxicity and enhance their effectiveness. Additionally, orally administered supplements are inevitably degraded by gastric acid, and this can influence their overall efficacy. In our previous study, we developed a convenient, pain-free, noninvasive, nondestructive, and needle-free supersonic spindle-flow atomizer for the transdermal delivery of microsized poly-L-lactic acid (PLLA) particles into the dermal layer of rat skin [21]. In this approach, supersonic gas is used to atomize a mixture solution, which enables even, efficient, and painless transdermal delivery of the solution into the dermal layer [21]. This enables the solution to rapidly and effectively enter capillary circulation, ensuring the supplements or drugs have their desired effects. In summary, the supersonic spindle-flow atomizer offers advantages over current techniques because it is noninvasive, nondestructive, painless, needle-free, and can be applied in situ.

The current study evaluated the efficacy of CSEs supplements using a novel approach—transdermal delivery using a supersonic atomizer. We investigated the association between grip strength declines and muscle damage induced by lambda carrageenan (LC) injection and exercise exposure in rats. In addition, we explored the reparative effects of transdermal pretreatment and post-treatment with CSEs as an alternative strategy for investigating antioxidant and nutritional supplementation.

## 2. Methods and Materials

The dried *Chaenomeles speciosa* fruit was purchased from a local store (Taiwan). H_2_O_2_ was purchased from Sigma, and 3–4,5-dimethylthiazol-2-yl-2,5-diphenyltetrazolium bromide (MTT), Dulbecco’s modified eagle medium (DMEM), fetal bovine serum, penicillin, and streptomycin were purchased from Gibco (Waltham, MA, USA). All the chemicals used in the present study were of analytical grade. The animal experiments conducted in this study were approved by the Institutional Animal Care and Use Committee of I-Shou University, Kaohsiung, Taiwan (IACUC-ISU-110-24, approval date: 30 June 2022).

### 2.1. Isolation and Characterization of CSEs

**Figure 1:**
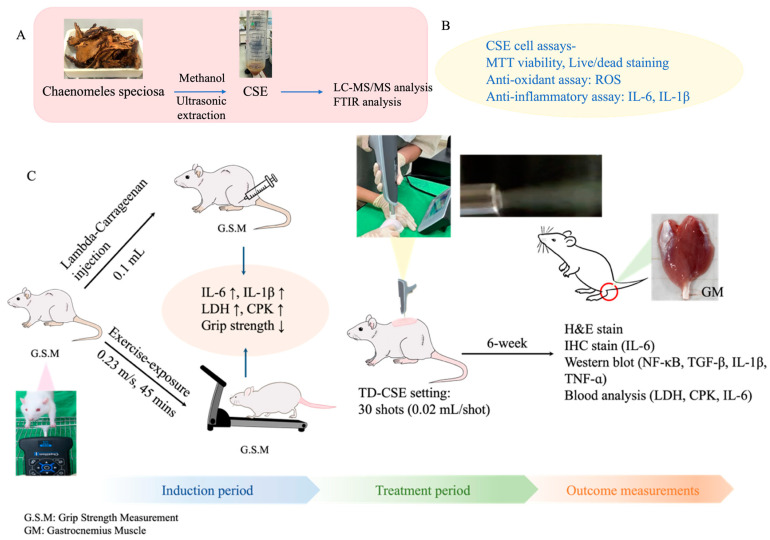

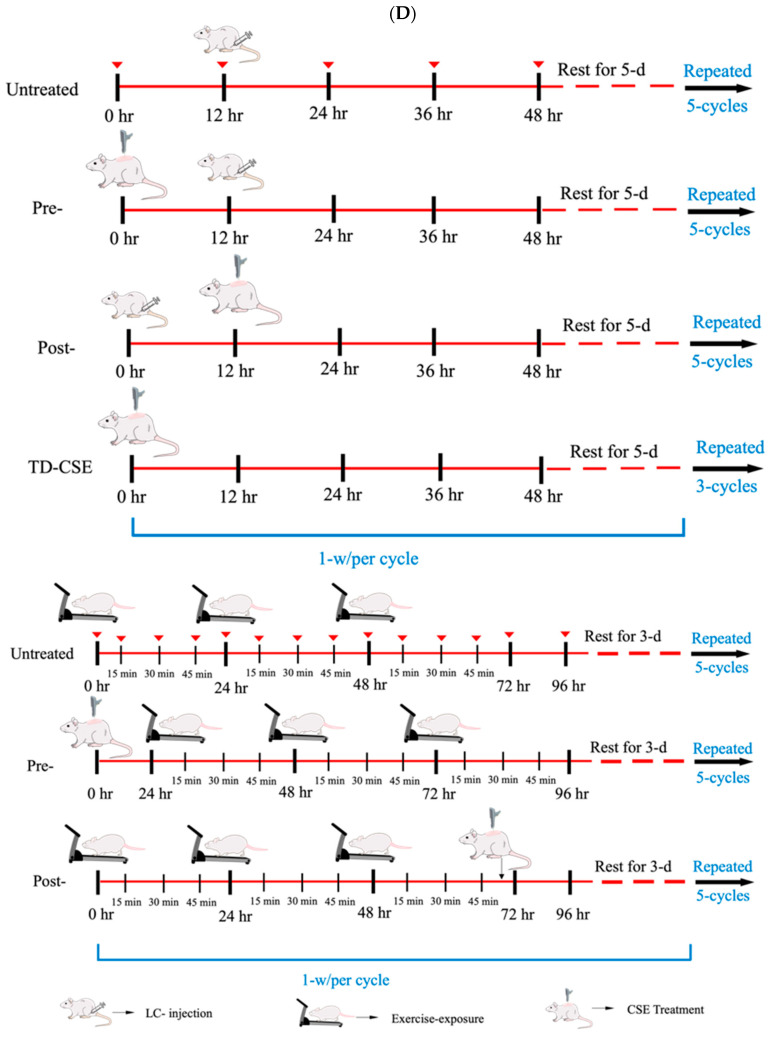
Schematic of (**A**) extraction process for *C. speciosa*, (**B**) in vitro cell tests for CSEs, (**C**) timeline and experimental design for in vivo animal studies and grip strength measurement, and (**D**) treatment procedures for rats subjected to LC injection (**upper** panel) and exercise (**lower** panel). G.S.M (red triangles): grip strength measurement.

The CSEs were extracted through ultrasonic oscillation (Delta, DC200H, Taiwan). In brief, 0.5 g of dried *C. speciosa* fruit was ground into a powder. It was then mixed with 25 mL of methanol and sonicated for 20 min (Figure 1A). After a 20 min extraction, the supernatant was collected and concentrated into a sticky paste by using a rotary evaporator (EYELA 1300VF, Japan). The resulting CSEs paste was stored at 4 °C for subsequent experiments. A Fourier-transform infrared (FTIR) spectroscopy analysis was performed to confirm the identity of the CSEs by comparing them with pure OA and UA purchased from Sigma. FTIR spectroscopy was employed to identify the characteristic peaks of the CSEs (Figure 1A).

The OA and UA analysis of the samples were conducted using an LC system coupled to a mass spectrometer (LCMS-8045, Shimadzu, Japan). An ACE C18 (5 μm, 250 × 4.6 mm) LC column (ThermoFisher, Waltham, MA, USA) was utilized to separate the OA and UA from the extracted sample matrix. The mobile phases were as follows: A—0.1% formic acid in water and B—methanol (99.9%) [22]. The flow rate was 0.5 mL/min at 32 °C in a column oven under gradient elution with a total analysis time of 70 min, using a sample injection volume of 5 μL. The mass spectrometer’s detection conditions included a CAD gas pressure of 320 kPa, nebulizing gas rate of 3 L/min, drying gas rate of 10 L/min, an ESI source, and DL line temperature of 300 °C; the heat block temperature was 350 °C. The detection was performed under the negative-ion mode (ESI−), with the selected ion monitoring (SIM) mode for the quantitative analysis at both 407 and 455 for the OA and UA; the results showed the presence of 1~2 ppm levels of OA and UA in the extracted samples (from a 100 mg sample extract).

### 2.2. In Vitro Viability Tests for CSEs

The C2C12 cells were cultured in high-glucose Dulbecco’s modified medium (DMEM, Gibco, NY, USA), supplemented with 10% fetal bovine serum (FBS) and 0.5% penicillin/streptomycin, at 37 °C in humidified air containing 5% CO_2_. When the cells were 80-85% confluent, they were subcultured and used to assess the effect of CSEs on the viability of the C2C12 cells; an MTT assay was performed. The C2C12 cells were seeded onto a 6-well plate at a density of 1.0 × 10^5^ cells/well and were allowed to attach for 24 h. Following attachment, the cells were treated with various concentrations of CSEs (5.25–21 mg/mL). After 24 h of treatment, 10 μL of MTT solution (5 mg/mL) was added to each well, and the plate was incubated for an additional 3 h. DMSO (Sigma) was added to each well to dissolve the formazan precipitate, and the absorbance of the formazan solution was measured at 450 nm by using a multiplate reader (Thermo Scientific, Waltham, MA, USA).

Cell viability was also assessed by using a live–dead cell assay (Invitrogen, Carlsbad, CA, USA). In brief, 1 mL of phosphate-buffered saline containing 2.5 μL/mL of 4 μM ethidium homodimer-1 (EthD-1) assay solution and 1 μL/mL of 2 μM calcein acetoxymethyl solution was prepared. This assay solution (100 μL) was added to the culture, and the mixture was incubated at 37 °C in a 5% CO_2_ incubator for 15 min. The sample was then observed using a fluorescence microscope at excitation wavelengths of 494 nm (green, calcein) and 528 nm (red, EthD-1).

### 2.3. Evaluation of Anti-Inflammatory Effects of CSEs on LPS-Induced C2C12 Cells

To evaluate the anti-inflammatory effects of CSEs on LPS-induced C2C12 cells, C2C12 cells (1.2 × 10^4^ cells/well) were seeded on a 24-well plate for 24 h and preincubated with 0.3 mg/mL LPS for 6 h [23]. Subsequently, the cells were treated with various concentrations of CSEs (0, 5.25, and 10.5 mg/mL). Following a 24 h incubation, the medium was collected and centrifuged at 1000× *g* for 20 min to obtain a cell-free supernatant (Thermo Scientific Fresco 17/21, Osterode am Harz, Germany). This supernatant was used in the enzyme-linked immunosorbent assay for IL-6 and IL-1β. The levels of IL-6 and IL-1β were quantified using an Elabscience Mouse IL-6 and IL-1β ELISA Kit (Minneapolis, MN, USA), and absorbance was measured at 450 nm in accordance with the manufacturer’s protocol.

### 2.4. Antioxidant Effect of CSEs

To assess the antioxidant effect of CSEs on H_2_O_2_-induced C2C12 cells, C2C12 cells (9 × 10^4^ cells/well) were seeded on a 6-well plate for 24 h and preincubated with 100 μM H_2_O_2_ for 3 h [24]. Subsequently, the cells were treated with various concentrations of CSEs (0, 5.25, and 10.5 mg/mL). Following a 24 h incubation, the ROS level was determined through dichlorodihydrofluorescein diacetate (DCFH-DA) staining in accordance with the manufacturer’s instructions (Elabscience, TX, USA). In brief, the cell suspension was mixed with diluted DCFH-DA reagent (10 μmol/L) and reacted at 37 °C for 30 min. The cells were then detected using a Fluoroskan FL Microplate Reader at excitation and emission wavelengths of 485 and 538 nm, respectively (Thermo Scientific).

### 2.5. Transdermal Delivery of CSEs through Needle-Free Supersonic Atomizer

A novel, needle-free, supersonic spindle-flow atomizer was developed on the basis of compressible flow theory [21]. In another study, we demonstrated that this spindle-flow nozzle can use low-pressure gas as a power source to generate a supersonic jet. This jet was then employed to atomize a hyaluronic acid solution containing PLLA microparticles, generating gas jets that facilitated the transdermal delivery of the PLLA microparticles across the skin barrier [21]. This supersonic atomizer is painless, needle-free, and noninvasive. In the present study, the settings for the atomizer in the in vivo animal study involving the transdermal delivery of CSEs (TD-CSEs) across the skin to the dorsal site of rats were as follows: inlet air pressure = 80 psi, number of shots = 30, and drug quantity delivered per shot = 0.02 mL (Figure 1C).

### 2.6. In Vivo Animal Experiments

#### 2.6.1. Establishment of Muscle Inflammation and Damage in Rat Model

The animal experiments were performed over 6 weeks (Figure 1C). A total of 22 Sprague Dawley rats purchased from BioLASCO, Co., Ltd., Taipei, Taiwan, aged 3 months at the onset of the experiments, were used to establish a rat model for muscle inflammation and damage. The rats were reared in a temperature- and humidity-maintained room and raised using a 12 h light–dark cycle (lights on at 6:00 a.m.). Food and water were offered ad libitum during the experiment. Additionally, two rats were designated as the healthy control group. Two distinct models of gastrocnemius muscle inflammation and damage in rats were developed: one was established through the injection of LC [15], and the other was established through 6 weeks of exercise exposure. In the LC-injection model group (injection of 0.1 mL of LC solution at a concentration of 3 mg/mL), the rats were anesthetized using Zoletil (intraperitoneal administration of 40 mg/kg of tiletamine with 50 mg/kg of zolazepam) and xylazine (10 mg/kg), and randomly assigned to the following groups (n = 3 per group). (1) Untreated group: Their grip strength was measured, and after that, LC was injected. The rats received no further treatment. Their grip strength was measured every 12 h. (2) Pretreatment group: Their grip strength was measured, and after anesthesia was administered, CSEs were transdermally delivered to the dorsal site by using a supersonic atomizer (30 shots). After a 12 h rest period, their grip strength was measured again. Subsequently, the rats were injected with an LC solution and allowed to rest for 12 h. Their grip strength was measured every 12 h. (3) Post-treatment group: Their grip strength was measured, and the rats were anesthetized and given an LC injection, which was followed by a 12 h rest period. Their grip strength was measured every 12 h. (4) TD-CSEs group (n = 1): Its grip strength was measured. After, anesthesia was administered, and CSEs were transdermally delivered, followed by a 12 h rest period. Its grip strength was measured every 12 h. The aforementioned experiments were conducted over 2 days and were followed by a 5-day rest period. This was repeated for five cycles (1 week per cycle; Figure 1D [upper panel]). In the exercise-exposure model, the rats underwent a physical training phase on a treadmill and were grouped as follows. (1) Untreated group: The grip strength of the rats was measured on day 1. This was followed by a 15 min run at a speed of 0.23 m/s, immediately after which the grip strength was measured. This exercise and measurement were repeated twice, at 30 and 45 min. After the exercise at 45 min, the rats were allowed to rest for 24 h, and the experiment was repeated at 24, 48, 72, and 96 h. (2) Pretreatment group: The grip strength of the rats was measured. After, anesthesia was administered, and CSEs were transdermally delivered to the dorsal side (30 shots). This was followed by a 24 h rest period, after which the grip strength was measured again. Subsequently, the rats were made to run for 15 min at a speed of 0.23 m/s, and the grip strength was measured again. The exercise and measurement were repeated twice, at 30 and 45 min. After the exercise at 45 min, the rats were allowed to rest for 24 h, and the experiment was repeated at 48, 72, and 96 h. (3) Post-treatment group: The experimental setting was similar to that for the untreated group before 72 h. After 72 h, the rats were anesthetized, CSEs were transdermally delivered to the dorsal side (30 shots). This was followed by a 12 h rest period, after which the grip strength was measured. The exercise-exposure experiments were conducted over 4 days, with a subsequent 3-day rest period. Control group: The grip strength was measured without LC injection or exercise exposure. This regimen was repeated for five cycles (1 week per cycle; Figure 1D [lower panel]). The activity, behavior, and appetite of the rats were carefully monitored twice per day. The rats were euthanatized by an overdose of CO_2_ after the 6-week experiment. The gastrocnemius muscle was harvested from the euthanized rats for histopathological and immunohistochemistry (IHC) analyses and Western blot staining.

#### 2.6.2. Histological Analysis

At the conclusion of the 6-week experimental period, the gastrocnemius muscles of the euthanized rats were harvested and fixed in 10% neutral-buffered formalin (Sigma, St. Louis, MO, USA). The resulting samples were then dehydrated in a graded ethanol solution, clarified in xylene (Sigma), embedded in paraffin blocks, and cut into 5 μm thick sections. A histopathological examination of the tissue samples was performed through hematoxylin and eosin (H&E) staining. The muscle fiber area after the CSEs treatments was determined from the longitudinal sections of the H&E stains by using ImageJ software (Version 1.50; National Institute of Health, Bethesda, MD, USA) (n = 3 images).

#### 2.6.3. Western Blot Analysis

To extract the total protein from both the treated and untreated gastrocnemius muscle samples, we employed a radioimmunoprecipitation assay buffer containing a phosphatase and protease inhibitor cocktail. The gastrocnemius muscle samples were incubated on ice for 60 min and centrifuged at 13,000× *g* and at 4 °C for 15 min. Following incubation, the protein concentrations in the supernatant were determined using a bicinchoninic acid protein assay kit. Subsequently, 40 μg of protein was loaded onto sodium dodecyl sulfate polyacrylamide gels and separated through electrophoresis. The separated proteins were then transferred onto polyvinylidene difluoride membranes. To prevent non-specific background binding of the primary and/or secondary antibodies to the membrane, the membranes were blocked in a milk-based blocking buffer (5% (*w*/*v*) non-fat dried milk in TBS with 0.1% (*v*/*v*) Tween 20) for 1 h, and incubated overnight with primary antibodies specific to NF-κB (1:500), TGF-β (1:500), IL-1β (1:1000), TNF-α (1:2000), and β-actin (1:500) at 4 °C. Finally, the membranes were incubated with enzyme-linked secondary antibodies at room temperature for 1 h. The levels of each protein were compared between the groups by using the semiquantitative intensity analysis function in ImageJ.

#### 2.6.4. IHC Analysis

For the IHC analysis, the sections were deparaffinized and rehydrated using graded concentrations of ethanol. Subsequently, the sections were treated with a hydrogen peroxide blocking solution for 10 min and washed with a phosphate-buffered solution. Then, the sections were subjected to heat treatment (at 95 °C) in a 0.01 M sodium citrate buffer with Tween 20. Each section was incubated with ImmunoBlock (PBS, pH 7.6, with 0.5% bovine serum albumin) at room temperature for 20 min and then washed with PBS. After, the sections were subjected to overnight incubation with rabbit antimouse IL-6 at 4 °C and then exposed to Mouse/Rabbit Probe HRP Labeling solution at 25 °C for 30 min. Finally, 3,3′-diaminobenzidine was applied for 10 min. The resulting intensity of the brown color, which indicated the presence of IL-6, was semiquantified using ImageJ software.

#### 2.6.5. Blood Biochemical Assays and IL-6 Inflammatory Factor Assay

The blood biochemical parameters, including lactate dehydrogenase (LDH) and creatine kinase (CK), were assayed to evaluate the recovery of muscle function following treatment with CSEs in the rats with muscle inflammation and damage induced by LC injection and exercise. Serum was extracted from the whole blood samples and used in the LDH and CK assays in accordance with the manufacturer’s instructions. The inflammatory factor IL-6 level was determined using an IL-6 assay kit in accordance with the manufacturer’s instructions.

### 2.7. Measurement of Forelimb Grip Strength

As described in our previous study [21], the grip strength of the rats was assessed using a standardized method, wherein each rat was lifted by the tail and prompted to grasp a rigid metal bar attached to a digital force gauge (AMETEK^®^, DFS3, Johnson Scale & Balance Co, NJ, USA). Subsequently, each rat was gently pulled backward by the tail until it released the metal bar. The reading displayed on the digital force gauge just before the rat released the metal bar was recorded as the grip strength. This assessment was conducted three consecutive times and the grip strength measurement for each rat is presented as the mean ± standard error of the mean.

### 2.8. Statistical Analysis

All the data are expressed as the means ± standard errors of the mean. Group differences were assessed using one-way analysis of variance (ANOVA) followed by Tukey’s multiple comparison test. A *p* value of <0.05 was considered significant. All statistical analyses were conducted using SPSS (version 20.0).

## 3. Results

### 3.1. Characterization of CSEs

**Figure 2:**
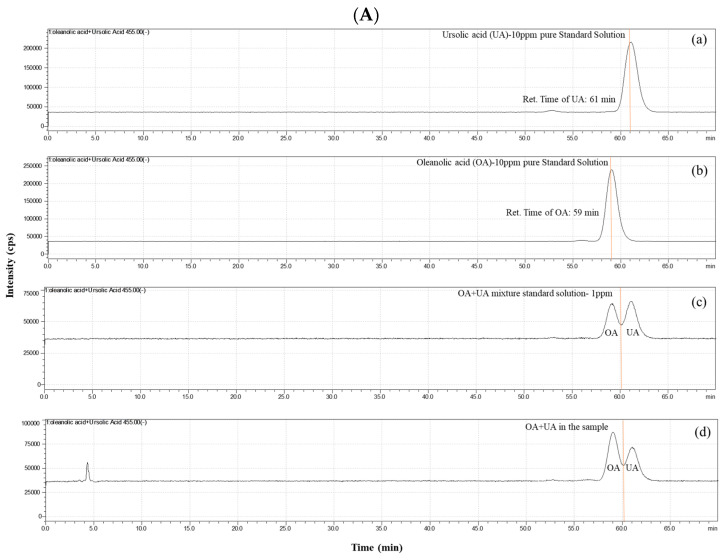

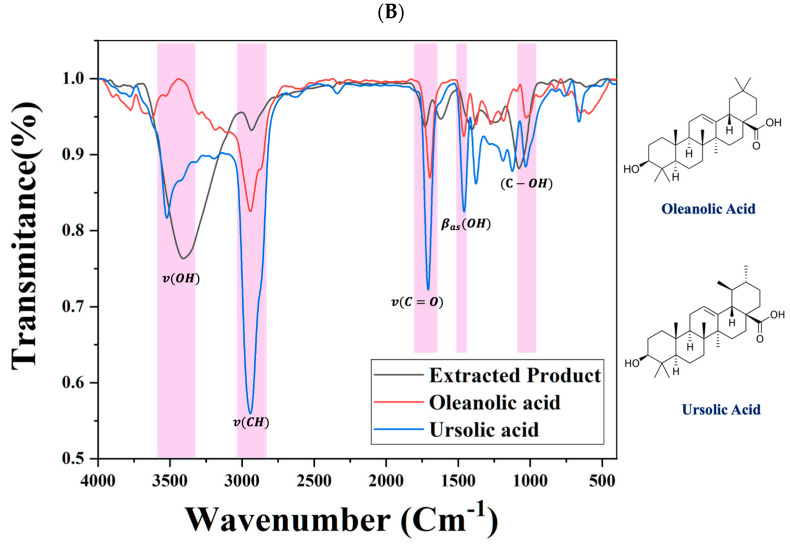
(**A**) Characterization of CSEs using LC-MS/MS: (**a**) standard TIC spectra for UA (at 10 ppm), (**b**) standard TIC spectra for OA (at 10 ppm), (**c**) standard TIC spectra for mixture of OA+UA (both at 1 ppm), and (**d**) extraction sample’s TIC spectra shows the presence of OA and UA. (**B**) Characterization of CSEs using FT-IR (blue line), UA ((standard chemical), red line), OA (standard chemical), and (**c**) extraction sample’s FT-IR spectra show the presence of OA and UA.

The present study employed ultrasonic sonication to extract CSEs, with OA and UA serving as the reference compounds to verify the composition of the CSEs. LC-MS/MS analyses revealed that the retention times for pure UA and OA were 61 and 59 min, respectively. The CSEs exhibited peaks at similar retention times, confirming the isolation of UA and OA from the CSEs (Figure 2A). The results showed the presence of 1 to 2 ppm levels of UA and OA in the extracted samples (from a 100 mg sample). The spectra of both molecules displayed a broad and redshifted νC-O band (around 1000~1050 cm^−1^), a νC=O band (around 1700~1750 cm^−1^), a νC-H band (around 2900~2950 cm^−1^), and a stronger νOH band (around 3450~3500 cm^−1^). These characteristic peaks closely resemble those of pure OA and pure UA, confirming the presence of these components in the CSEs (Figure 2B).

### 3.2. Effects of CSEs on Viability and Anti-Inflammatory and Antioxidant Properties of C2C12 Cells

**Figure 3:**
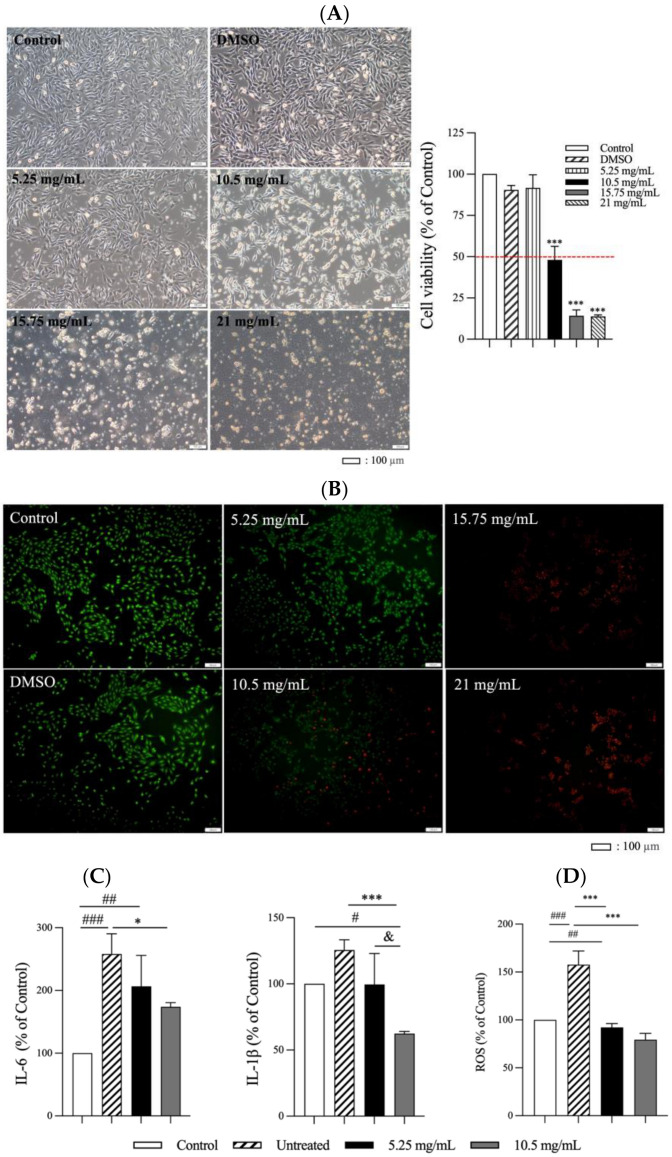
Cell morphologies and results of the MTT assay (magnification: 100×) conducted on C2C12 cells treated with various concentrations of (**A**) CSEs. *** *p* < 0.001, compared with the control group. (**B**) Live/dead staining of C2C12 cells treated with CSEs. Green indicates live cells, whereas red indicates dead cells. (**C**) Anti-inflammatory effects on LPS-induced C2C12 cells treated with CSEs (5.25 and 10.5 mg/mL). (**D**) Effect of CSEs on the reduction in ROS regeneration induced by H_2_O_2_ in C2C12 cells. * *p* < 0.05, *** *p* < 0.001, compared with the untreated group. # *p* < 0.05, ## *p* < 0.01 and ### *p* < 0.001, compared with the control group. & *p* < 0.05 5.25 mg/mL compared to 10.5 mg/mL. The above tests were performed after a 24 h culturing period.

The results of the MTT assay (Figure 3A) indicate that treatment with increasing concentrations (5.25–21 mg/mL) of CSEs for 24 h led to a reduction in cell viability. The IC_50_ was approximately 10.5 mg/mL. The morphological changes observed in the treated cells included the retraction of cell pseudopodia, rounding, and detachment from the culture surface, indicating that higher CSEs concentrations led to an increased amount of cell death. A similar trend in cell viability was also observed in the live/dead staining (Figure 3B).

LPS, at a concentration of 0.3 mg/mL, was employed to induce a significant inflammatory response in C2C12 cells. To ensure non-cytotoxicity to the C2C12 cells, we used concentrations equivalent to half the IC_50_ of CSEs, and assessed the concentrations’ anti-inflammatory and antioxidant effects. The anti-inflammatory effects of CSEs on C2C12 cells with LPS-induced inflammation are depicted in (Figure 3C). Treatment with CSEs at concentrations of 5.25 and 10.5 mg/mL significantly suppressed the levels of inflammatory factors IL-6 and IL-1β. Notably, the higher concentration of CSEs (10.5 mg/mL) more effectively suppressed the inflammatory response than the lower concentration of 5.25 mg/mL did when the responses were compared with those from the untreated group.

Cell exposure to 100 μM H_2_O_2_ for 1 h resulted in increased ROS generation (Figure 3D). However, this increase was significantly reduced following treatment with CSEs. Notably, treatment with 10.5 mg/mL of CSEs led to a higher reduction in ROS generation.

Considering cell cytotoxicity and the observed range of the anti-inflammatory and antioxidant effects of CSEs on the C2C12 cells, we used a CSEs concentration of 5.25 mg/mL in the subsequent in vivo animal experiments. These experiments were conducted to assess the treatment’s effects on the rats with muscle inflammation induced by LC injection and exercise.

### 3.3. Muscle Inflammation or Damage Induction and Histological Phenotyping

**Figure 4:**
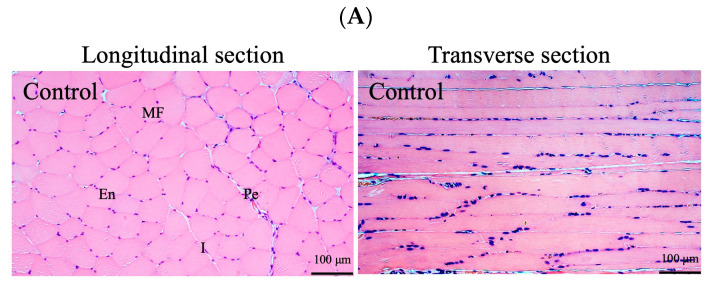

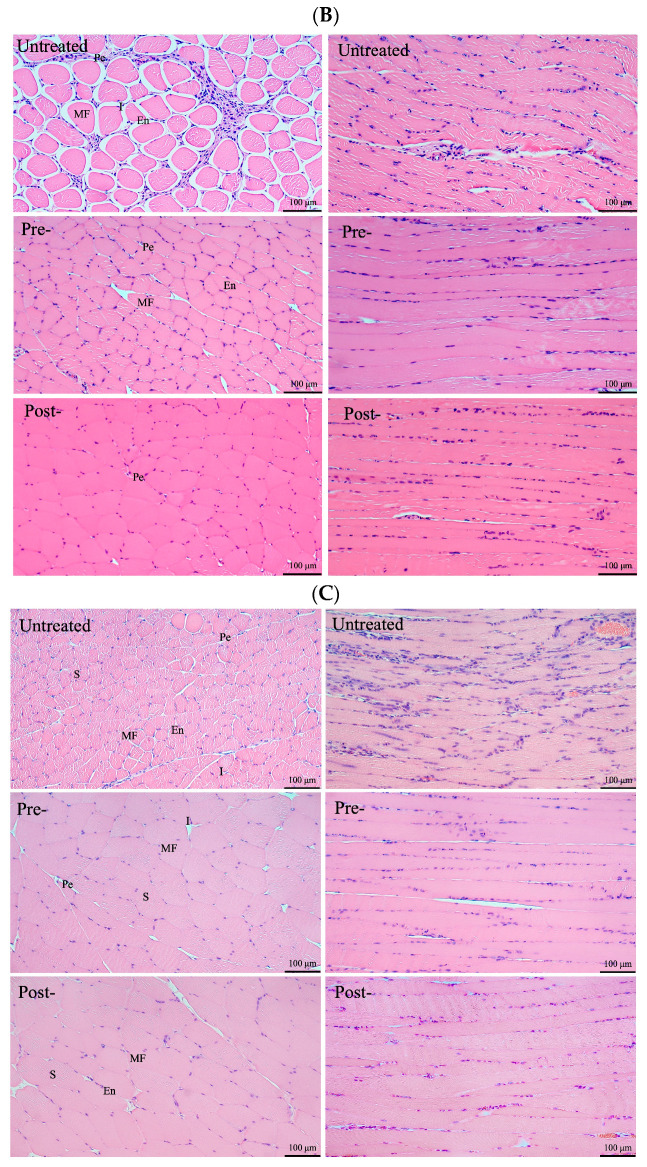

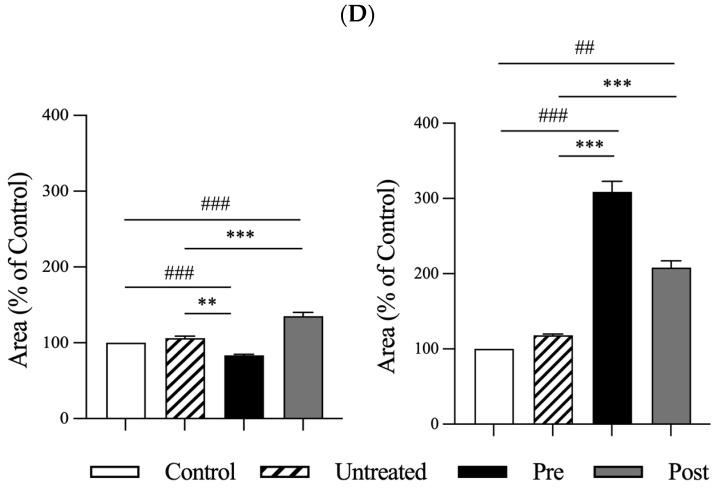
H&E staining of gastrocnemius muscle. (**A**) Normal control group; (**B**) LC-injection group; (**C**) exercise-exposure group, and (**D**) the area of muscle fibers determined from longitudinal section images (n = 3). Left panel: LC-injection group; right panel: exercise-exposure group. The result indicate the CSEs treatment exerted a positive restorative effect on the fiber size and morphology of the muscle. Untreated: induction with treatment; Pre-: transdermal delivery of CSEs into the rats before induction; Post-: transdermal delivery of CSEs into the rats after induction. ** *p* < 0.01, *** *p* < 0.001, compared with the untreated group. ## *p* < 0.01 and ### *p* < 0.001, compared with the control group (n = 3 per group). MF: muscle fiber; Pe: perimysium; En: endomysium; S: splitting; I: inflammation; V: vascularization.

An evaluation of the gastrocnemius muscle following LC injection and 6 weeks of exercise exposure revealed distinct histological changes (Figure 4). The healthy control rat’s muscle sections exhibited normal polygonal fiber shapes and were devoid of inflammation or splitting; the muscle fibers appeared striated, and the endomysium and perimysium were normal sized (Figure 4A). By contrast, the muscle sections from the LC-injected rats exhibited severe infiltration of the inflammatory cells, multiple macrophages between muscle fibers, a noticeable increase in the perimysium and endomysium gap, and the muscle fibers exhibited splitting and reduced striation (Figure 4B). The rats in the LC-injected group pretreated (pretreatment group) with CSEs for 6 weeks exhibited considerably less inflammation, their muscle fibers returned to a polygonal shape, and their muscle fibers appeared more striated, but had some splitting. For the muscle fibers from the LC-injected group post-treated (post-treatment group) with CSEs, notable recovery changes were noted in the muscle histology, with reduced or no inflammation, striated muscle fibers, and less splitting in the fibers observed. These results indicate that both pretreatment and post-treatment with CSEs could mitigate the inflammatory response induced by LC injection, leading toward the recovery of the gastrocnemius muscle’s normal histology.

The H&E stains of the gastrocnemius muscles from the exercise-exposure groups are presented in Figure 4C. In the rats subjected to exercise, noticeable alterations in the muscle fiber architecture were observed, including an increased gap in the perimysium and endomysium, prominent splitting in the muscle fibers, the infiltration of inflammatory cells, and enhanced vascularization (untreated group). Both pretreatment and post-treatment with CSEs significantly mitigated the inflammatory response, reducing the perimysium to a much smaller size, comparable to that of the normal control group. The muscle fibers regained a highly polygonal fiber shape and a striated appearance, and reduced splitting and a null endomysium were observed (Figure 4C, pretreatment and post-treatment groups). Notably, the area of the muscle fibers in the rats exposed to LC injection and exercise after CSEs treatment was significantly greater than that in the untreated rats (Figure 4D). Overall, CSEs treatment exerted a positive restorative effect on muscle morphology in the rats from both exposure groups.

### 3.4. Western Blot Analysis

**Figure 5:**
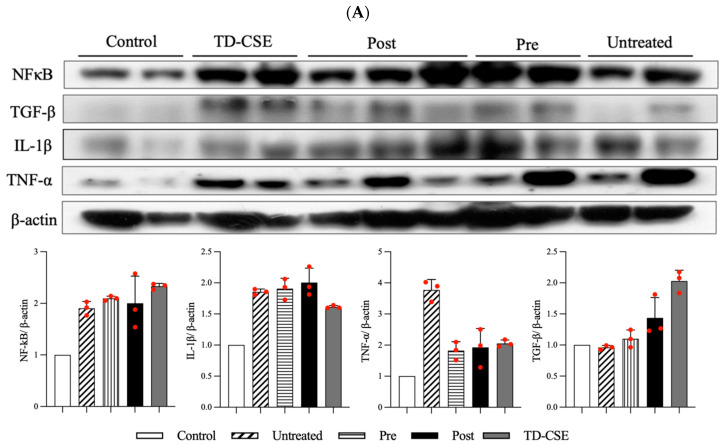

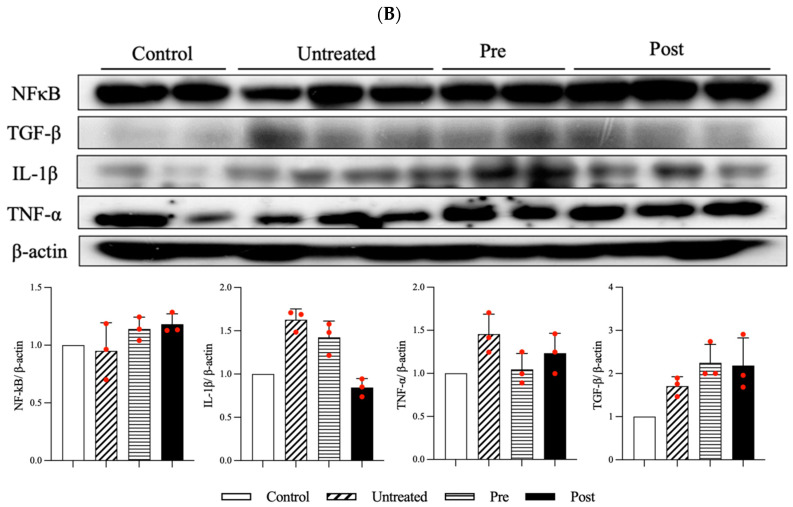
Results of Western blot analysis and semiquantitative assay of NF-κB, IL-1β, TNF-α, and TGF-β for two models: (**A**) LC-injection model and (**B**) exercise-exposure model. An increase in NF-κB levels was noted in the pretreatment and post-treatment groups, indicating that these treatment strategies activated the subsequent inflammatory response to aid in the repair of damaged muscle. Reduced levels of IL-1β and TNF-α, compared with those for the untreated group, demonstrate an anti-inflammatory capability after treatment with CSEs. Conversely, TGF-β levels increased, similarly indicating the onset of muscle regeneration or repair after CSEs treatment (n = 2).

Inflammatory responses in musculoskeletal tissues play a crucial role in tissue pathogenesis. The NF-κB is a key signaling factor implicated in inflammation and mediates the upregulation of TNF-α and IL-1β activities in muscle repair [25]. TGF-β, an influential anti-inflammatory cytokine, mitigates the inflammatory effects of TNF-α and IL-1β. Studies have highlighted the importance of TGF-β for the regeneration of damaged muscle, particularly after eccentric muscle contraction [26]. After 6 weeks of treatment, we harvested the rat gastrocnemius muscles and isolated the total proteins for a Western blot analysis of NF-κB, TNF-α, IL-1β, and TGF-β levels. In the LC-injection muscle damage model, the NF-κB levels considerably increased in the untreated, pretreatment, and post-treatment groups compared with the control group (Figure 5A). This elevation in NF-κB levels after LC injection indicates an upregulation of subsequent inflammatory responses and the healing process. The TNF-α levels were considerably decreased in the CSEs pretreatment and post-treatment groups, whereas the IL-1β levels were within a similar range across the untreated, pretreatment, and post-treatment groups. We additionally investigated the effects of TD-CSEs administration on normal rats (procedure shown in Figure 1D). The results revealed that the TD-CSEs group exhibited increased levels of NF-κB and reduced levels of IL-1β and TNF-α compared with the untreated, pretreatment, and post-treatment groups. Because they are a foreign reagent, the CSEs induced an inflammatory response in the rats. However, they demonstrated an anti-inflammatory capability after the acute inflammation period, resulting in lower levels of inflammatory factors. Conversely, the TGF-β levels increased, indicating the onset of muscle regeneration or repair after CSEs treatment, with the TGF-β level being highest in the TD-CSEs group. These findings collectively suggest that CSEs can exert anti-inflammatory effects and contribute to the repair of muscle damaged through LC injection.

Studies have indicated that prolonged or regular exercise training can lead to the onset of an inflammatory response associated with leukocyte activation, muscle function deterioration, and a notable decline in muscle strength [27]. However, this inflammation is essential for the regeneration and repair of muscles and, therefore, numerous studies have investigated the role of nutritional and antioxidant supplementation in modulating oxidative stress and inflammation associated with exercise. As depicted in Figure 5B, an increase in NF-κB levels was noted in the pretreatment and post-treatment groups, indicating that these treatment strategies activated the subsequent inflammatory response to aid in the repair of damaged muscle. Notably, the post-treatment group, which received transdermal delivery of CSEs, exhibited a considerable decrease in IL-1β levels relative to those in the untreated and pretreatment groups. Although the TNF-α levels were lower in the pretreatment group, CSEs treatment led to a reduction in TNF-α levels, indicating that CSEs alleviated the inflammatory response in the rats after exercise exposure. Moreover, the TGF-β levels were higher in the CSEs-treated groups than in the untreated group, indicating the greater regeneration and repair of muscle. Notably, the untreated group also exhibited an elevated TGF-β expression relative to that in the control group that can likely be explained by the natural regeneration and repair processes that occur following exercise (Figure 5B).

### 3.5. IHC and Serum Analysis for IL-6

**Figure 6:**
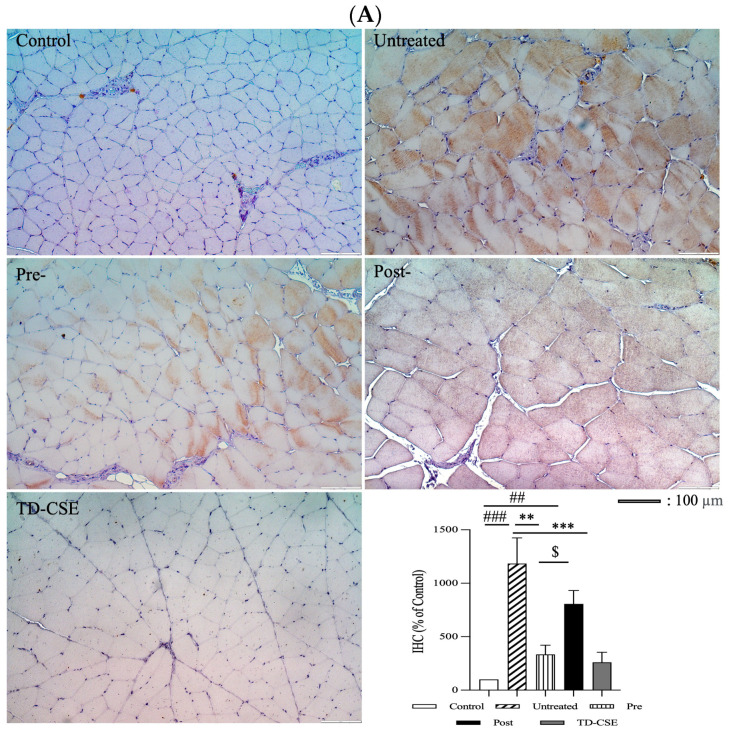

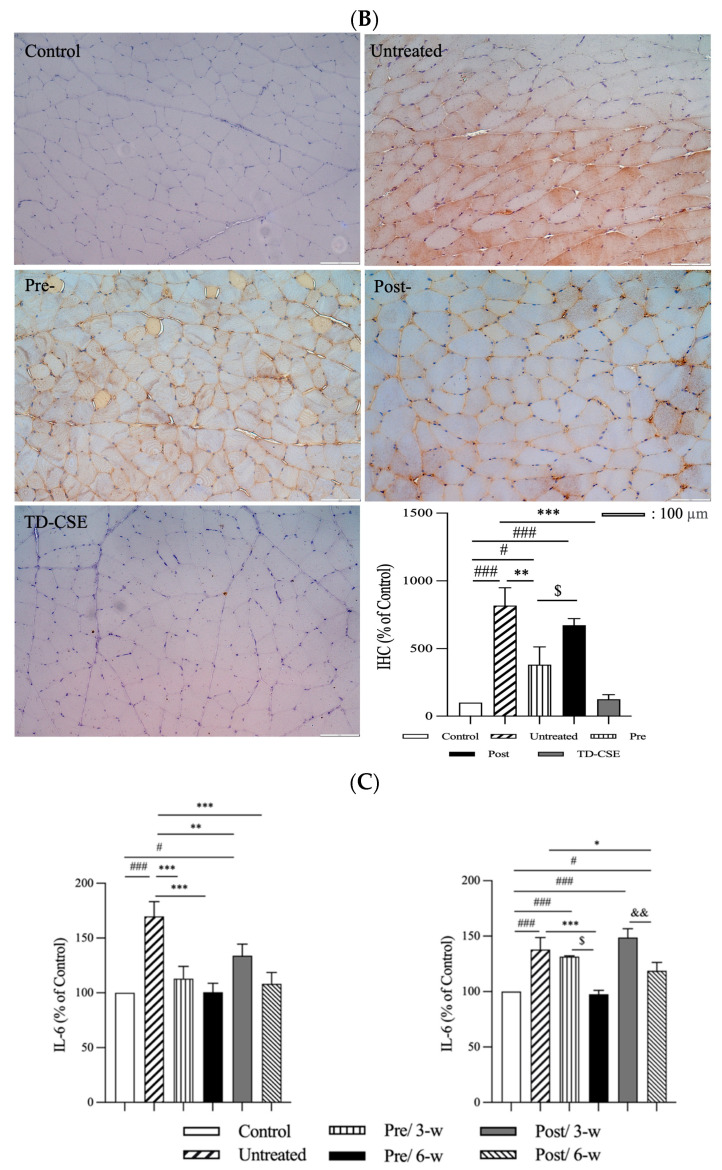
Results of IHC staining and semiquantitative analyses for (**A**) LC-injected rats (magnification: 200×). Semiquantitative analysis of IL-6 staining between groups (n = 3 images) of (**B**) rats exposed to exercise. (**C**) Serum analysis of IL-6 (left panel: rats exposed to LC injection; right panel: rats exposed to exercise). * *p* < 0.05, ** *p* < 0.005, *** *p* < 0.001, compared with the untreated group. # *p* < 0.05, ## *p* < 0.01, and ### *p* < 0.001, compared with the control group. $ *p* < 0.05, pre- compared with the post-group. && *p* < 0.005, post/3 w compared with the post/6 w group.

We employed an IHC and serum analysis to detect IL-6 production after treatment. In the LC-injection model, the gastrocnemius muscle treated with CSEs exhibited a less intense brown color, indicating lower levels of IL-6 compared with those for the untreated group (Figure 6A). The group that received CSEs pretreatment (pretreatment group) exhibited the lowest IL-6 staining intensity, followed by the CSEs post-treatment and untreated groups. The untreated group displayed higher IL-6 production, with notable infiltration of the inflammatory cells. By contrast, the normal rat treated with CSEs (TD-CSEs group) exhibited nearly no brown staining indicating IL-6. As illustrated in Figure 6A, we semiquantitatively determined the IHC IL-6 staining intensity after CSEs treatment. The inflammation reaction was alleviated after the CSEs treatments and, notably, the CSEs pretreatment group exhibited the lowest IL-6 staining intensity, indicating that anti-inflammatory effects were exerted by the CSEs on the muscle. The IL-6 staining results indicate that CSEs are able to reduce the inflammatory response in the LC-injection muscle.

A similar reduction in IL-6, as determined through staining, was observed in the rats exposed to exercise that underwent CSEs treatment (Figure 6B). The group pretreated with CSEs (pretreatment group) exhibited more pronounced anti-inflammatory activity than did the other groups. This enhanced anti-inflammatory effect may be attributed to the pretreatment with CSEs, which could have reduced ROS regeneration, subsequently suppressing the inflammatory response. Notably, fewer inflammatory cells were present between the muscle fibers. Furthermore, the inflammatory response induced by exercise exposure, which can be considered a form of chronic inflammation, was suppressed by CSEs. In the normal rats treated with CSEs (TD-CSEs), a similar expression of IL-6 was observed, indicating that CSEs do not induce an obvious immune or inflammatory response.

Serum samples were collected at 3 and 6 weeks. As indicated in Figure 5 CSEs pretreatment and post-treatment both had anti-inflammatory effects when the pretreatment and post-treatment groups were compared with the untreated group of rats injected with LC, and 6 weeks of CSEs treatment enhanced the anti-inflammatory response and reduced IL-6 levels (Figure 6C, left panel). In the rats exposed to exercise, CSEs pretreatment resulted in enhanced anti-inflammation effects when the pretreatment group was compared with the post-treatment and untreated groups. Furthermore, a prolonged 6-week exercise exposure along with CSEs pretreatment and post-treatment resulted in a significant decrease in IL-6 levels.

### 3.6. Serum Analyses for LDH and CK

LDH is a vital enzyme in the anaerobic metabolic pathway and is widely distributed in almost all tissues, occurring in particularly high concentrations in the muscle, liver, and kidneys. LDH is also a nonspecific marker of tissue turnover, a normal metabolic process. LDH assays enable the measurement of leaked LDH in the serum from damaged tissues. During strenuous exercise, LDH activity increases and generates lactic acid under normal physiological conditions, which leads to serum LDH levels being elevated in cases of muscle injury. In the field of sports medicine, LDH holds potential as an indicator of muscle responses to training, with LDH observably increasing in skeletal and cardiac muscles within 3–5 h post-training [28]. Because it is widespread, LDH can serve as a marker for various tissue injuries. Furthermore, a decrease in LDH levels during treatment can serve as an indicator of a better prognosis or a positive response to treatment. CK is commonly regarded as the most reliable indirect marker of muscle tissue damage, particularly after resistance exercise or activities involving predominantly eccentric actions [29]. Different types of muscle contractions, including concentric, eccentric, and static actions, have the potential to inflict damage on muscle tissue [30]. Both resistance and aerobic exercises induce CK and LDH changes that increase muscle damage postexercise [31]. The extent of these changes appears to be influenced by the specific exercise protocol and the individual’s training status [32]. As indicated in Table 1, the LDH values in the control group for the rats exposed to LC injection and exercise increased gradually from week 1 to week 6, but remained within the normal range [28]. By contrast, the LDH values gradually increased to an abnormal range (>400 U/L) at 6 weeks, indicating tissue injury after repeated LC injections. Additionally, the CK values in the untreated group reached abnormally high levels in the LC-injected rats at 6 weeks. Notably, the LDH values significantly decreased and returned to the normal range (<200 U/L) after 6 weeks of transdermal delivery of CSEs, in both the pretreatment and post-treatment groups. The degree of change in CK was influenced by the duration and type of exercise, with strenuous exercise resulting in more pronounced elevations. The observed increase in CK values in the current study can be attributed to damage to the muscle fiber structures. As shown in Table 1, the CK values increased with time in the untreated groups, indicating progressive muscle damage. Overall, these findings suggest that CSEs treatment effectively mitigates muscle damage by reducing elevated LDH and CK levels induced by LC injections and exercise exposure.

### 3.7. Grip Strength Measurement

**Figure 7:**
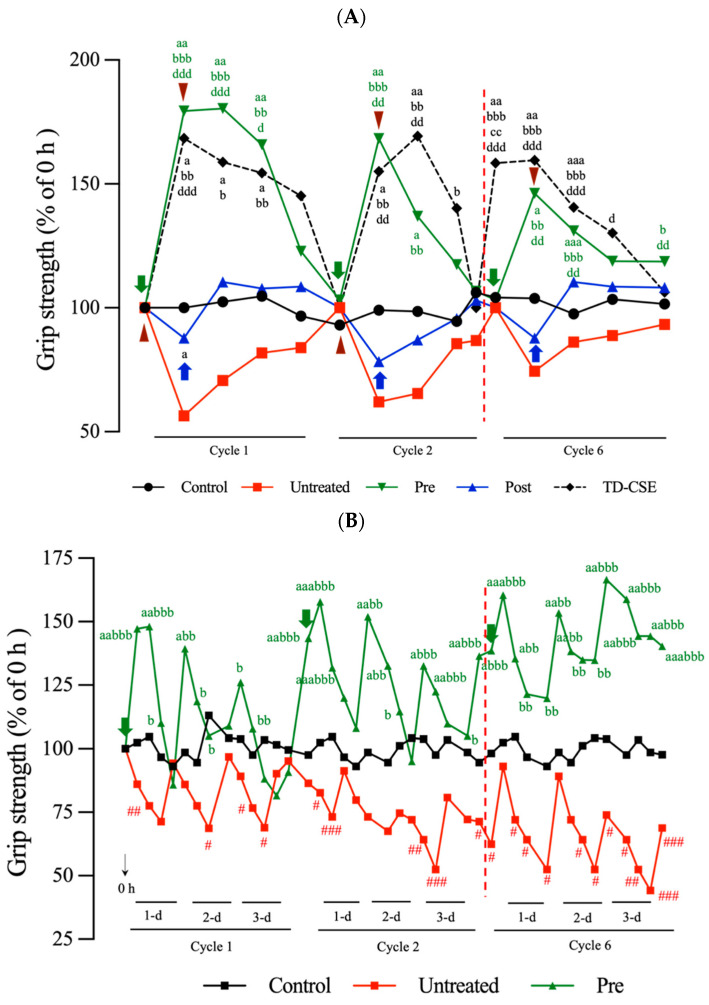

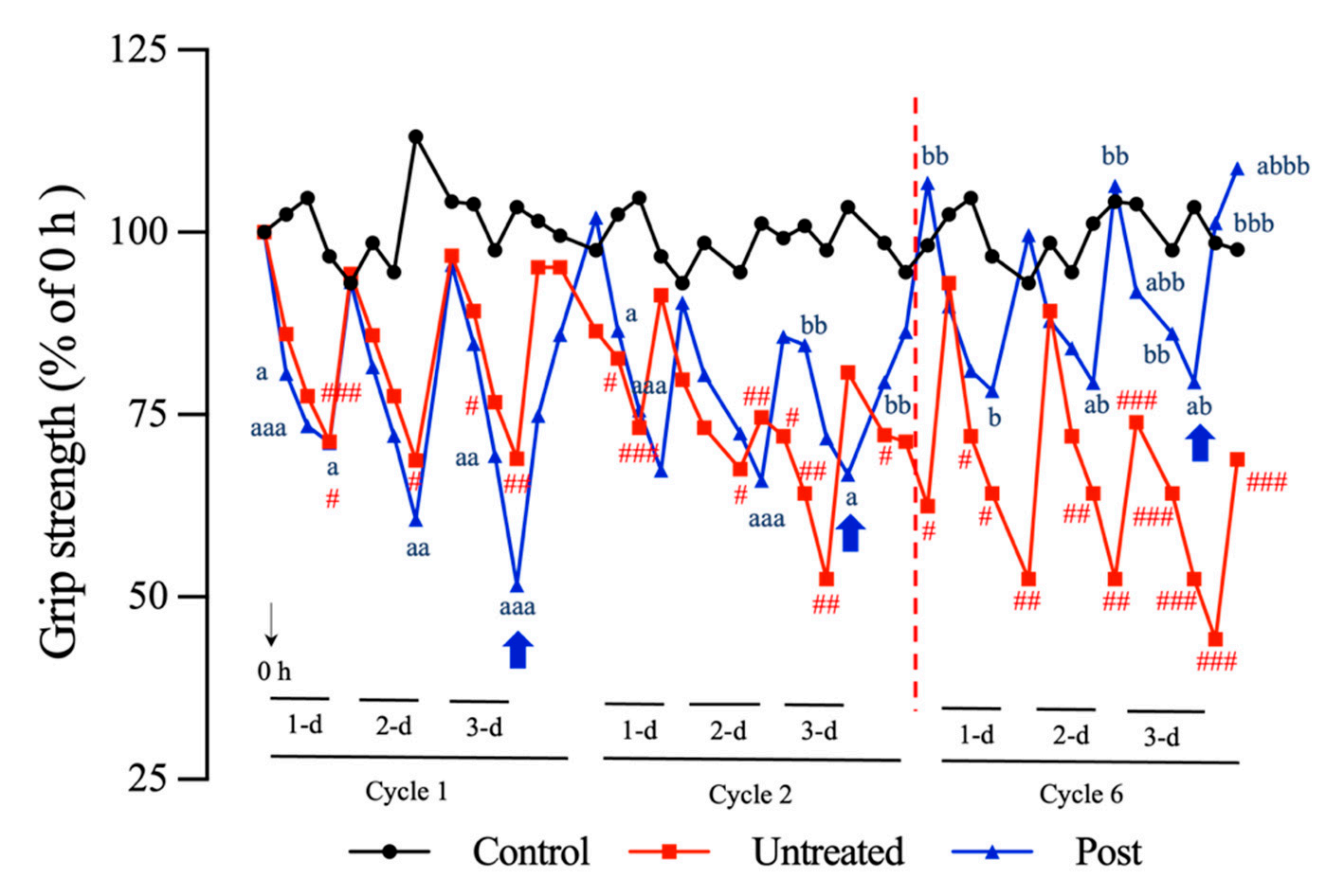
Changes in grip strength in (**A**) LC-injected rats and (**B**) exercise-exposure rats before and after treatment with CSEs. Brown triangles indicate times of LC injections, green arrows indicate times of pretreatment with CSEs, and blue arrows indicate times of post-treatment with CSEs. ^a^ *p* < 0.05, ^aa^ *p* < 0.01, and ^aaa^ *p* < 0.001, pre-group compared with the control group; ^b^ *p* < 0.05, ^bb^ *p* < 0.01, and ^bbb^ *p* < 0.001, pre-group compared with the untreated group; ^d^ *p* < 0.05, ^dd^ *p* < 0.01, and ^ddd^ *p* < 0.001, pre-group compared with the post-group; ^a^ *p* < 0.05, ^aa^ *p* < 0.01, and ^aaa^ *p* < 0.001, TD-CSEs group compared with the control group; ^b^ *p* < 0.05, ^bb^ *p* < 0.01, and ^bbb^ *p* < 0.001, TD-CSEs group compared with the untreated group; ^cc^ *p* < 0.01, TD-CSEs group compared with the pre-group; ^d^ *p* < 0.05, ^dd^ *p* < 0.01, and ^ddd^ *p* < 0.001, TD-CSEs group compared with the post-group; ^#^ *p* < 0.05, ^##^ *p* < 0.01, and ^###^ *p* < 0.001, untreated group compared with the control group; ^a^ *p* < 0.05, ^aa^ *p* < 0.01, and ^aaa^ *p* < 0.001, post-group compared with the control group; ^b^ *p* < 0.05, ^bb^ *p* < 0.01, and ^bbb^ *p* < 0.001, post-group compared with the control group (n = 3 per group). Red dotted lines represents Cycle 3 to Cycle 5 are skipped.

The grip strength test was conducted to assess the effects of CSEs pretreatment and post-treatment on gastrocnemius muscle function in the rats subjected to LC injections and exercise over 6 weeks. In the LC-injection group, the rats injected with 0.1 mL of LC solution (indicated by the brown triangles in Figure 7A) experienced a significant reduction in grip strength of approximately 55% relative to that of the control group (untreated group in Figure 7A). Their grip strength was gradually restored after the first cycle of treatment (1 week per cycle), because of the effects of normal metabolic process on LC. After the subsequent LC injections, similar patterns of grip strength changes were observed in the subsequent cycles. Both pretreatment and post-treatment with the transdermal delivery of CSEs resulted in a significant increase in grip strength relative to that of the untreated group. In the pretreatment group (pretreatment with CSEs before LC injection), CSEs induced a rapid and significant increase in grip strength, with the grip strength reaching 180% of that of the control group within the initial 12 h, and remaining high at 24 h. The grip strength gradually decreased over time, eventually returning to a similar range as that of the normal group. Conversely, in the post-treatment group (post-treatment with CSEs after LC injection), the grip strength initially decreased after LC injection, increased after CSEs treatment, and subsequently remained higher than that of the control group. These results indicate that CSEs have the potential to enable resistance to the effects or damage caused by LC, and facilitate the repair of damaged muscle, leading to the restoration of grip strength. Notably, in the normal rats that were transdermally treated with CSEs, the grip strength significantly increased by approximately 165% at 12 h post-treatment. The grip strength gradually decreased and returned to levels comparable to those of the control group.

In the exercise-exposure groups, the grip strength exhibited a decline after each 15 min period of exercise exposure, reaching approximately 75% of the initial grip strength measured at 0 h (cycle 1). After a 1-day rest period, the grip strength was restored to levels comparable to those of the control group. However, in the untreated group, the grip strength exhibited a declining trend over the six cycles of exercise exposure (Figure 7B, upper panel). As was true with the rats exposed to LC injection, the rats pretreated with CSEs (pretreatment group) before undergoing exercise exposure exhibited an immediate and significant increase in grip strength of approximately 150% after 30 min of exercise, relative to the grip strength of the control group (cycle 1). Notably, the grip strength exhibited an increasing trend over six cycles of exercise exposure in the CSEs pretreatment group. However, the rats treated with CSEs after exercise exposure exhibited a different response; the grip strength of the post-treatment group exhibited a declining trend with each exercise exposure, with recovery occurring after a 1 d rest period. The grip strength declined again after the subsequent exercise exposures. On day 3 postexposure, the grip strength significantly increased, reaching levels similar to those of the control group after CSEs treatment (blue arrow in Figure 7B, lower panel). This pattern of change in grip strength persisted across all six cycles of the repeated operations. Notably, an increasing trend in grip strength was observed after additional treatments of CSEs were administered, even after the rats received exercise exposure (Figure 7B, lower panel). These results indicate that pretreatment with CSEs led to a more favorable and substantial increase in grip strength compared with post-treatment. This could be attributed to the CSEs actively preventing and reducing the generation of ROS and scavenging the ROS generated during exercise. Additionally, pretreatment with CSEs might have protected against and alleviated the onset of the inflammatory response induced by exercise, although inflammation aids in muscle regeneration. Post-treatment with CSEs led to a delayed and less pronounced increase in grip strength, indicating that CSEs acted more passively as a scavenger of the generated ROS, alleviating inflammation and attenuating LDH production in the rats exposed to exercise. In the rats exposed to LC injection, the grip strength of the control group ranged from 1.47 ± 0.22 to 1.69 ± 0.14 N, that of the untreated group ranged from 0.82 ± 0.08 to 1.45 ± 0.11 N, that of the pretreatment group ranged from 1.40 ± 0.24 to 2.48 ± 0.13 N, that of the post-treatment group ranged from 1.39 ± 0.26 to 1.95 ± 0.12 N, and that of the TD-CSEs group ranged from 1.29 ± 0.14 to 2.18 ± 0.05 N. In the rats exposed to exercise, the grip strength of the control group ranged from 1.48 ± 0.16 to 1.79 ± 0.22 N, that of the untreated group ranged from 0.85 ± 0.12 to 1.91 ± 0.13 N, that of the pretreatment group ranged from 1.23 ± 0.09 to 2.51 ± 0.07 N, and that of the post-treatment group ranged from 1.11 ± 0.06 to 2.35 ± 0.03 N. Furthermore, the pretreatment groups demonstrated a better recovery of grip strength compared with the untreated group, and had greater grip strength than the control group did, indicating an improvement in muscle function in the pretreatment groups.

## 4. Discussion

Studies have investigated the effectiveness of various physiotherapeutic, nutritional, and pharmacological approaches for mitigating muscle damage, particularly intramuscular inflammation, and enhancing strength postexercise [33,34,35]. The results of these studies have been conflicting and inconclusive with respect to the benefits of such strategies at mitigating muscle damage. In the present study, we investigated the effects of pretreatment and post-treatment with CSEs delivered transdermally by using a supersonic atomizer on grip strength decline and muscle damage resulting from LC injection and exercise exposure in rats. The triterpenoids OA and UA were isolated from *C. speciosa* through ultrasonic sonication. Although CSEs exhibit mild cytotoxicity at concentrations exceeding 10.5 mg/mL, they have potent antioxidant and anti-inflammatory effects on C2C12 cells at concentrations of 5.25 and 10.5 mg/mL. As indicated in Figure 3, the levels of inflammatory factors IL-6 and IL-1β, as well as ROS generation, were significantly reduced following CSEs treatment. Furthermore, we explored the use of CSEs as an ergogenic antioxidant and anti-inflammatory agent that could simultaneously repair damaged muscle and restore grip strength in rat models. Notably, we evaluated the supplementation by using painless, needle-free transdermal delivery through an atomizer.

Intense exercise increases ROS production and muscle inflammation. Although a helpful inflammatory response occurs after exercise, muscle damage during activity can adversely affect muscle strength. Furthermore, a prolonged inflammatory response can partially impede muscle regeneration. The present study observed notable histological changes in the rats subjected to LC injection and exercise exposure. The gastrocnemius muscles in these rats underwent a notable change in muscle fiber architecture, with an increased endomysium and perimysium gap, pronounced splitting, a less striated appearance, and enhanced vascularization (Figure 4). The splitting phenomenon became more prominent after repeated exposure to exercise [36]. Studies have reported that extreme and prolonged exercise can lead to muscle splitting, muscle regeneration, myocyte grafting, and pronounced hypertrophy, indicating that splitting might contribute to muscle recovery by forming new fibers and increasing the cross-sectional area of muscle fibers [37]. In the present study, evident fiber splitting was observed in the gastrocnemius muscles of the untreated groups. However, after treatment with CSEs, low fiber splitting but higher hypertrophy, expressed in the cross-sectional area of the fibers, were observed (Figure 4D). This indicates that CSEs may improve the environmental conditions for muscle growth or regeneration, possibly by reducing ROS regeneration and the inflammatory response between muscle fibers. This increased fiber area was likely partially responsible for the improvement in grip strength noted in the treatment groups in this study (Figure 7).

An inflammatory response to exercise commonly occurs during strenuous exercise, and is accompanied by muscle damage, leukocyte activation, infiltration of the inflammatory cells, the release of inflammatory mediators, and fibrinolysis. In the current study, the histological observations revealed the obvious infiltration of the inflammatory cells and macrophages between the muscle fibers in both the LC-injected and exercise-exposure groups (Figure 4B,C; untreated groups). Previous findings have indicated that IL-1β plays a role in tissue injury, and the infiltration of mononuclear cells has a greater impact on muscle repair than on tissue damage. Furthermore, pro-inflammatory mediators, such as TNF-α and IL-6, are released in response to exercise, with the intensity of the exercise influencing the resulting levels of these mediators [38]. In the present study, higher levels of the inflammatory factors IL-1β and TNF-α were observed in the untreated groups, indicating that the muscles were in an inflamed state, possibly due to the infiltration of the inflammatory cells and muscle injury (Figure 5). After treatment with CSEs, the IL-1β and TNF-α levels were considerably decreased, indicating that CSEs alleviated the inflammatory responses. Studies have proposed that an increase in IL-1β, TNF-α, and IL-6 may indicate tissue injury or worsened tissue injury, particularly when an individual has prolonged exercise exposure, and a persistent increase in these factors may contribute to later fibrotic responses [39]. IL-6 levels tend to increase more than those of other cytokines during exercise, and such increases might indicate muscle damage [40]. A persistent elevation of IL-6 is associated with muscle atrophy, which can lead to a reduction in strength and muscle function and an increase in muscle pain [41]. In the current study, the untreated groups exhibited substantially higher levels of IL-6 (Figure 6); CSEs treatments significantly reduced IL-6 expression, indicating the alleviation of inflammation and injury.

We hypothesized that grip strength declines could be associated with muscle inflammation caused by LC injection and long-term exercise, and such declines were observable immediately after the rats received exercise exposure. Progressive and greater declines were observed as the duration of exercise exposure increased in the untreated group; however, grip strength recovered during the rest period (Figure 7). A slower recovery in grip strength was noted in the LC-injected rats. However, CSEs were able to reduce ROS generation and exert anti-inflammatory effects on the inflamed muscle, thereby establishing better environmental conditions for muscle repair or regeneration. The data for this study indicate that grip strength is inversely correlated with the levels of inflammatory factors IL-1β, TNF-α, and IL-6. Pretreatment with CSEs rapidly increased grip strength and progressively enhanced it over time (6 weeks). However, in the LC-injected rats, although CSEs treatment increased grip strength and led to a sustained increase in grip strength, the grip strength gradually returned to levels comparable to those of the control group within 6 weeks. These differences in grip strength changes between the exercise-exposure and LC-injection groups may be attributable to the greater extent of splitting of muscle fibers during exercise, yielding a larger fiber area after spontaneous muscle repair and CSEs treatment (Figure 4D), which may have resulted in an increased grip strength.

In summary, our in vivo animal study demonstrated the following benefits of CSEs transdermally delivered by a supersonic atomizer.

1. CSEs can reduce the inflammatory response caused by LC injection and exercise exposure.
2. Pretreatment with CSEs can prevent inflammation and significantly increase grip strength.
3. Patented supersonic atomization provides painless, needle-free, and in situ features that can be used to promote the performance of muscles.

**Figure 8:**
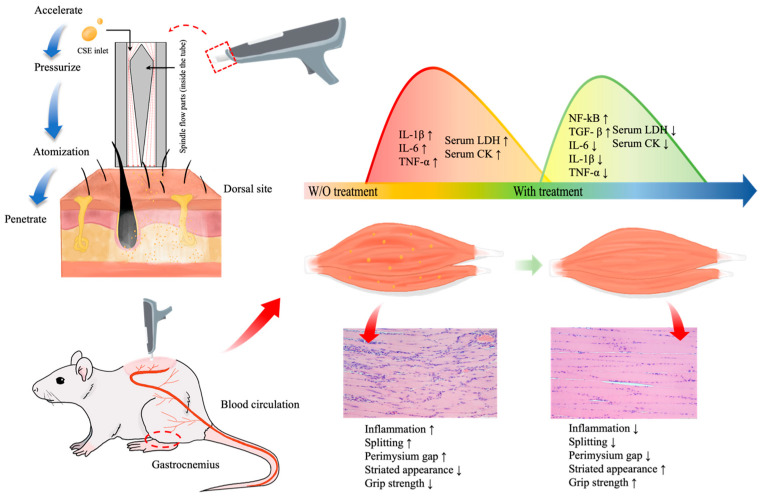
Schematic of the atomizer design and effects of CSEs on gastrocnemius muscle. CSEs treatment led to a reduction in inflammation factors, restoration of muscle histology, and increase in grip strength in LC-injected and exercise-exposure rats.

A schematic of the design of the atomizer and the conclusive impacts of CSEs supplements on the inflammatory response, histological alterations, and grip strength are presented in Figure 8. The comprehensive data indicate that pretreatment with CSEs leads to a more efficient reduction in inflammatory responses, increased grip strength, and improved muscle repair. These effects are confirmed through the histological analysis, the identification of significant changes in the Western blot analysis, IHC results, and observed enhancements in muscle grip strength.

The limitations of this study include the selection of animal species, the small sample size, the non-direct application of CSEs onto the muscle, and the short follow-up time. A larger animal species, larger sample size, and longer follow-up may be required to study the usefulness of CSEs for sports applications in the near future.

## 5. Conclusions

CSEs can reduce the inflammatory response by reducing the levels of inflammatory factors IL-1β, TNF-α, and IL-6 in the gastrocnemius muscle. Furthermore, CSEs contributed to the repair of damaged muscle and significantly enhanced grip strength, indicating that they can improve muscle function after training or participation in a competition. In this study, pretreatment with CSEs resulted in a progressive and greater increase in grip strength over a 6-week period. Furthermore, the transdermal delivery of CSEs by using a supersonic atomizer helped circumvent the digestive processes in the stomach, thereby enhancing the efficacy of CSEs. This painless, needle-free, and in situ supersonic spindle-flow atomizer is a promising innovation in sports medicine.

## Figures and Tables

**Table 1:** Serum analyses of LDH and CK (n = 1).

LDH (U/L)	Control	Untreated	Pre	Post
Lambda Carrageenan injection				
1 W	143	371	448	371
2 W	242	445	392	299
6 W	290	686	201	66
Exercise exposure				
1 W	143	315	380	327
2 W	242	420	223	312
6 W	290	463	182	94
CK (U/L)	Control	Untreated	Pre	Post
Lambda Carrageenan injection				
1 W	171	635	363	998
2 W	175	998	210	302
6 W	237	1026	156	90
Exercise exposure				
1 W	171	467	315	409
2 W	175	591	258	353
6 W	237	697	170	123

## Data Availability

The data that support the findings of this study are available within this article.
